# New Remedy for Strangury

**Published:** 1846-09

**Authors:** 


*New Remedy for Strangury.*—The Editor of the Buffalo Medical Journal, referring to Dr. Gordon's remedy for Strangury, reported in this Journal, Nov. 1845, *an infusion of the honey bee*, says.

"The following new remedy will not strike the reader as a very prepossessing addition to the materia medica. We have been informed, however, by a practitioner of this city, that in a case of strangury in which the introduction of the catheter was attempted without success, the prescription was tried with immediate relief; and it was subsequently repeated daily, with the same results, until the occasion for its administration ceased."

